# Discovering New G-Quadruplex DNA Catalysts in Enantioselective Sulfoxidation Reaction

**DOI:** 10.3390/ijms23031092

**Published:** 2022-01-20

**Authors:** Carmen Festa, Veronica Esposito, Daniela Benigno, Simona De Marino, Angela Zampella, Antonella Virgilio, Aldo Galeone

**Affiliations:** Department of Pharmacy, University of Naples Federico II, Via D. Montesano 49, I-80131 Naples, Italy; carmen.festa@unina.it (C.F.); verespos@unina.it (V.E.); daniela.benigno@unina.it (D.B.); sidemari@unina.it (S.D.M.); angela.zampella@unina.it (A.Z.); galeone@unina.it (A.G.)

**Keywords:** catalytic DNA, telomeric G-quadruplex analogues, asymmetric synthesis, sulfoxidation

## Abstract

The natural human telomeric G-quadruplex (G4) sequence d(GGGTTAGGGTTAGGGTTAGGG) HT21 was extensively utilized as a G4 DNA-based catalytic system for enantioselective reactions. Nine oligonucleotides (ODNs) based on this sequence and containing 8-bromo-2′-deoxyadenosine (A^Br^), 8-oxo-2′-deoxyadenosine (A^oxo^) or β-L-2′-deoxyadenosine (A^L^) at different single loop positions were investigated to evaluate their performances as DNA catalysts in an enantioselective sulfoxidation reaction of thioanisole. The substitution of an adenosine in the loops of HT21 with these modified residues had a negligible impact on the G4 DNA structural features, thermal stability, and catalytic activity, since almost all investigated ODNs were able to form G-quadruplexes strictly resembling that of HT21 and catalyze a full conversion of the thioanisole substrate. More marked effects were obtained in chiral selectivity of G4 DNA metalloenzymes, considering that in most cases the DNA-modified catalysts induced lower enantioselectivities compared to the natural one. However, the HT21 derivative containing an A^L^ residue in the first loop sequence significantly proved to be capable of producing about 84% enantiomeric excess, the highest enantioselectivity for DNA-based oxidation reaction to date.

## 1. Introduction

DNA-based asymmetric catalysis has recently attracted increasing attention, thus becoming a particularly interesting tool for organic chemical synthesis. The double-stranded DNA (dsDNA) has been regarded as a catalytic species and applied in various key asymmetric reactions [1,2,3,4]. The right-handed chiral conformation of dsDNA plays a key role in enantioselective catalysis, making this secondary structure a promising chiral inducer [1,2,3,4]. A DNA-based catalyst comprises a non-chiral ligand that can chelate a transition metal ion; therefore the catalyzed reaction takes place in, or very close to, the DNA helix to allow the chirality of DNA to be transferred onto the reaction [1,2,3,4]. The DNA double helix provides the chiral microenvironment to selectively form one enantiomer of a given reaction product. Several studies have been carried out to highlight the mechanisms by which the chirality is transferred and the influence of the interaction between DNA and the metallic co-factor on the selectivity [5]. Furthermore, taking advantage of the unique structure of L-DNA, left-helical enantioselective induction has been obtained in different DNA-based asymmetric catalyzed reactions, thus allowing tuning the absolute configuration in DNA-based asymmetric catalysis, predicting access to both enantiomers for any reaction [6].

Recently, G-quadruplex DNA structures (G4 DNA) met with growing success in catalysis, with these structures able to induce sensible levels of enantioselectivity in several asymmetric reactions. Indeed, the concept of G4 DNA-based catalysts was first introduced in 2010 [7]. Since then, G4 DNA have been applied in asymmetric Diels–Alder and Friedel–Crafts reactions [8,9]. Furthermore, G4 DNA were employed to speed up the transformation in aldol reaction [10] and sulfoxidation [11], confirming that the G4 DNA could also act as a suitable chiral template.

G4 DNA are one of the alternative conformations that guanine-rich DNA strands can adopt [12]. G-rich nucleic acid sequences are inclined to form four-stranded structures (G4), in which a G-tetrad is stabilized by Hoogsteen hydrogen bonds [13]. G-quadruplexes can readily form in solutions containing monovalent cations, specifically K^+^ and Na^+^.

**Figure 1:**
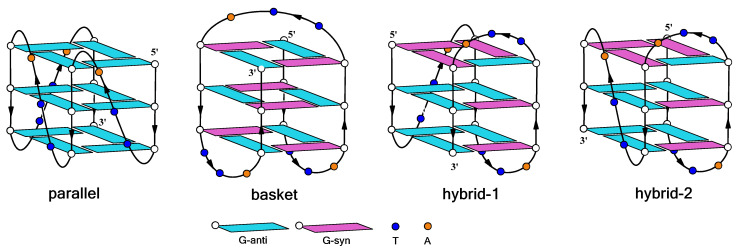
Schematic representation of G-quadruplex topologies formed by HT21 in different buffer solutions.

Unlike dsDNA, G4 DNA possess a remarkable structural variability, depending on the strand molecularity (tetra-, bi-, monomolecular) or arrangement (parallel, antiparallel, etc.), the presence and type of the loops (propeller, lateral, diagonal), the glycosidic conformation (*syn* or *anti*) of the G-residues, and the groove sizes [12]. Furthermore, the conformation of G4 DNA can be self-regulated by the external environment. For example, the human telomeric G-quadruplex sequence d(GGGTTAGGGTTAGGGTTAGGG) HT21 can form anti-parallel, parallel, or hybrid-type monomolecular G-quadruplex conformations in different buffer solutions [14,15] (Figure 1). The remarkable structural variability of G4 DNA constitutes a valuable resource to design new DNA-based catalysts, usable in synthetic organic chemistry. In turn, the study of the relationship between DNA structure topology and catalytic reaction performances could be useful to recognize and clarify the different features that influence the stereo-induction.

The catalytic performance of the G-quadruplexes can be influenced by several factors, among them, the topology of the quadruplex and the nature of the ligand, which can be non-covalently or covalently linked to the secondary structure, thus tuning the stereoselectivity in DNA-based asymmetric catalysis [16,17].

G-quadruplexes show two peculiar structural features: a central core formed by stacked G-tetrads complexing a K^+^ ion (mostly accounting for the structural stability) and, in general, some loops protruding outwardly, composed by one to four nucleotides. The G-tetrad planar arrangement plays a very important role in recognizing planar aromatic ligands through stacking interactions; therefore, in most cases, the organic reaction occurs on the upper or lower G-tetrads of the DNA-based catalyst. Loop residues adjacent to the terminal G-tetrads in specific conformations provide the special environment of the organic reaction; so, their sequences could affect both the reaction rate and the enantiospecificity. 

The natural telomeric G-quadruplex HT21 has been extensively utilized as G4 DNA-based catalytic system for enantioselective Diels–Alder [8,18,19,20] reactions, while, to the best of our knowledge, few studies have been reported on DNA-based enantioselective sulfoxidation reaction [11,21]. Enantiomerically pure sulfoxides have interesting potentials both in pharmaceutics, as biologically active compounds [22], and in asymmetric synthesis, as chiral ligands and organocatalysts [23]. The main method to obtain enantiopure sulfoxides is the enantioselective oxidation of sulfide. Most of the biological oxidation reactions are usually catalyzed by protein enzymes and cofactors in water [23,24]. The first DNA-based enantioselective sulfoxidation reaction was achieved in 2016 [11] with an achiral Cu(II)-4,4′-bimethyl-2,2′-bipyridine complex non-covalently interacting with G4 HT21, using H_2_O_2_ as oxidizing agent. The authors found that the G-quadruplex helix backbone is responsible for the catalytic enantioselectivity and activity; indeed, the chirality of DNA can be transferred into the sulfoxides, providing up to 77% enantiomeric excess. A later study, aimed to characterize the interactions of Cu(II)-bipyridine cofactors and thioanisole substrates with HT21, proved that bipyridine complexes showed nonspecific binding properties and the thioanisole interacted with G-quadruplex structure at around the second loop and 3′-end, confirming that the reaction usually occurs on a terminal G-tetrad in a space surrounded by the loop residues [21]. It is generally known that a human telomeric G4 DNA metalloenzyme is able to influence the configuration and the enantioselectivity of the reaction products and that the loop sequence in the G4 DNA catalyst has a high influence on chiral expression [9]. 

**Figure 2:**
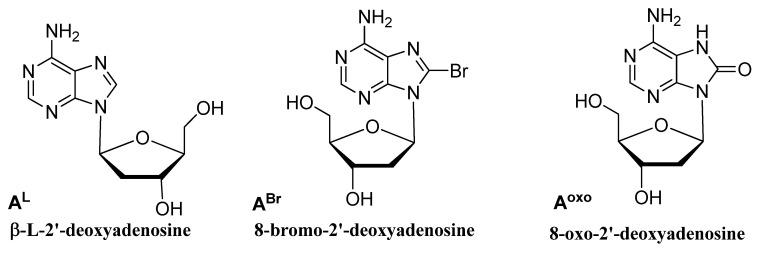
Chemical structures of the adenosine derivatives introduced in different loop positions of HT21.

In order to explore the role of the residues in the loops and to improve the performances of G4 DNA catalysts, a series of HT21 analogues were prepared, in which each sequence contained a chemically modified monomer replacing, one at a time, natural adenosines in the TTA loops. Since minor changes in the loop sequences of G4 DNA can have notable consequences on the chiral orientation of the enantioselective reactions [9], straightforward chemical modifications were made to single adenosine loop residues in close proximity to the upper or lower G-tetrad, by using commercially available adenosine derivatives (Figure 2). Specifically, HT21 derivatives containing 8-bromo-2′-deoxyadenosine (A^Br^), 8-oxo-2′-deoxyadenosine (A^oxo^), and β-L-2′-deoxyadenosine (A^L^) were prepared (Table 1), and the effect of different loop sequences on the activity and the enantioselectivity of G4 DNA metalloenzyme in the sulfoxidation reaction were tested. These modified nucleosides were chosen either to evaluate the electronic and steric effects of different groups in the C-8 position of loop adenines being part of the microenvironment surrounding and orienting the substrate, or to take advantage of an L-DNA residue to transfer chirality and modulate the enantioselectivity.

## 2. Results

### 2.1. CD Spectroscopy

CD spectra of the modified HT21 were acquired and compared with their natural counterpart in the same buffer and temperature conditions of the reaction (see below). CD is a very suitable technique for the characterization of G4 forming oligonucleotides, particularly, to obtain information concerning the G-quadruplex folding topology, considering that several G4 types show distinctive CD spectra [25,26]; therefore, a straightforward comparison of the CD profiles of HT21 and its derivatives can be useful in identifying similar topological features. 

**Figure 3:**
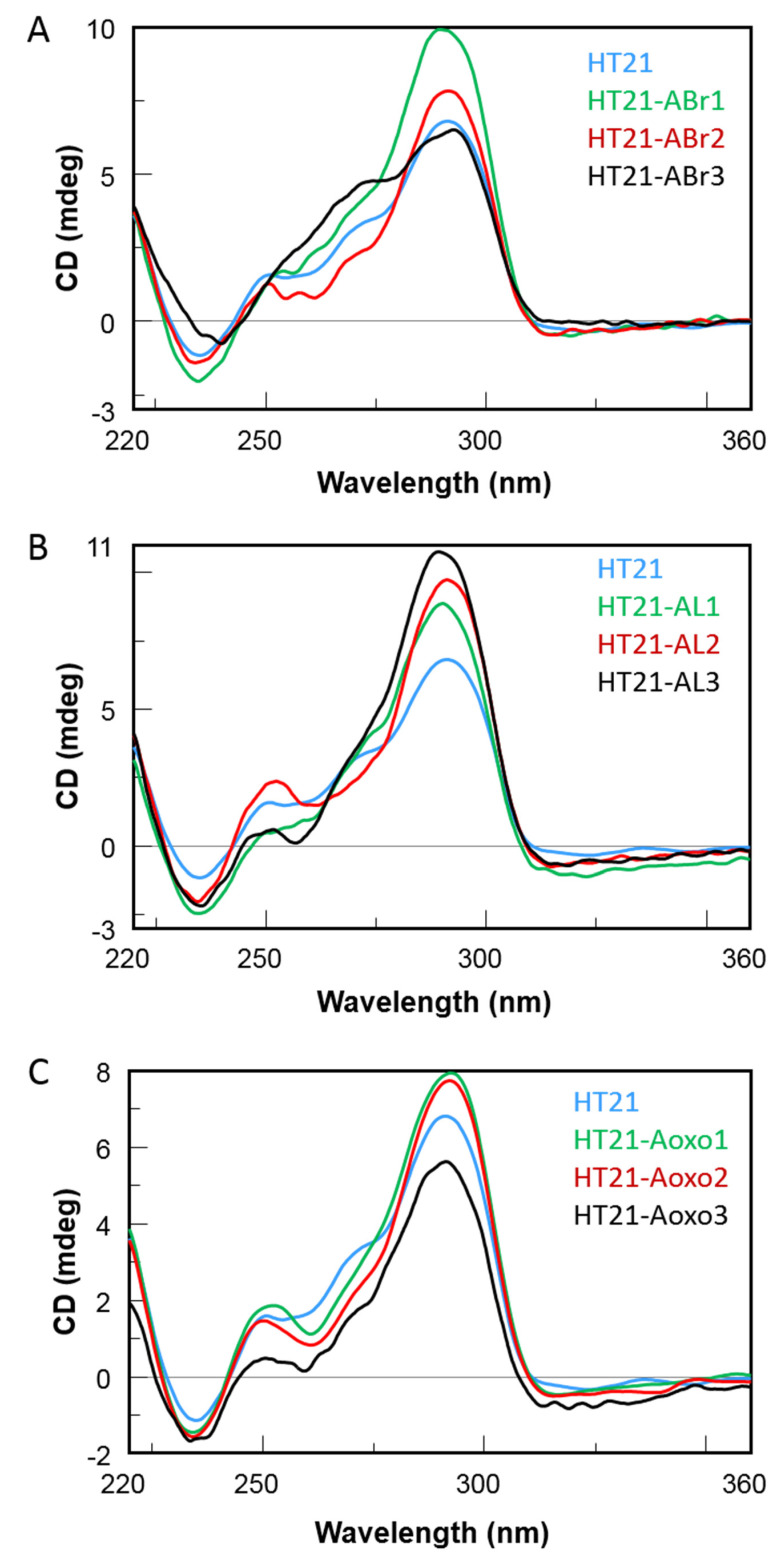
CD spectra of HT21 and its A^Br^ (Panel **A**), A^L^ (Panel **B**) and A^oxo^ (Panel **C**) derivatives in 20 mM MOPS buffer (pH 7.0) containing 150 mM KCl (20 μM in ODN).

The CD profile of natural telomeric sequence HT21 showed a positive peak around 290 nm, a major shoulder at 270 nm, and a smaller one at 250 nm with a small negative peak around 240 nm, accordingly with hybrid-type telomeric G-quadruplex structures [27]. Regarding HT21-A^Br^-modified sequences, HT21-ABr1 and HT21-ABr2 displayed CD profiles very similar to each other and to their natural counterpart, apart from slight differences in band intensity around 290 nm, thus indicating that the ability to adopt hybrid-type G-quadruplexes is preserved. More significant differences were observed for HT21-ABr3 CD spectrum in the shoulder region around 250–270 nm, thus suggesting the presence of other G-quadruplex conformations in addition to the hybrid ones (Figure 3A).

Concerning the spectra of the HT21-A^L^-modified G4, their CD profiles confirmed a strong positive peak around 290 nm, peculiar of the hybrid-type telomeric G-quadruplex [27], and a minor positive one at 250 nm, while the shoulder around 270 nm was less marked or completely absent (HT21-AL2) (Figure 3B).

Finally, HT21-A^oxo^ derivatives showed CD profiles almost superimposable to each other, particularly HT21-Aoxo1 and HT21-Aoxo2, and closely comparable to that of the natural one, apart from the region around 270 nm, as in some previous cases (Figure 3C).

The whole of the data clearly indicated that none of these derivatives folded into a G4 parallel structure containing all-*anti* G residues (whose typical CD spectrum was characterized by a major positive band at 260 nm and a negative peak at 240 nm) but rather the hybrid-type G-quadruplex of the natural telomeric sequence was very likely to be the main conformation adopted by them, despite tiny structural dissimilarities supposedly involving the modified loop regions.

In order to estimate the effect of the introduction of modified residues on the G4 thermal stability, HT21 derivatives were subjected to CD melting experiments in comparison with their natural counterpart, under the same experimental conditions. All the measurements were performed at an ODN concentration of 20 µM in 20 mM MOPS buffer (pH 7.0) containing 150 mM of KCl. The melting profiles of the modified sequences were quite similar to that of the natural sequence (Figure S1 in Electronic Supplementary Materials). Most of the HT21 analogues showed melting temperatures strictly comparable to or slightly higher than the natural one (Table 1), thus revealing that the substitution of an adenosine in the loops of HT21 with the evaluated modified residues has a negligible impact on the G4 DNA thermal stability.

### 2.2. Catalytic Properties of HT21 Analogues

**Scheme 1:**
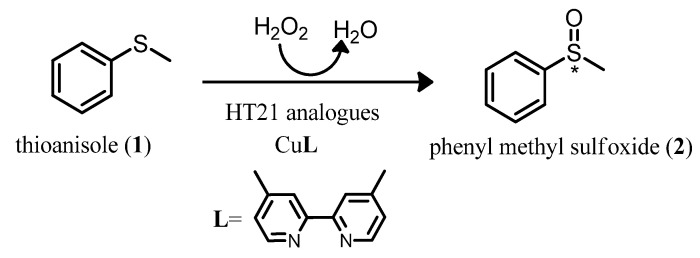
Enantioselective sulfoxidation reaction of thioanisole (**1**) with Cu(II)-4,4′-bimethyl-2,2′ bipyridine/HT21 analogues catalysts to obtain phenyl methyl sulfoxide (**2**).

To investigate the catalytic properties of HT21 derivatives, the enantioselective sulfoxidation reaction, catalyzed by human telomeric G4 DNA and Cu(II) complexed with 4,4′-bimethyl-2,2′-bipyridine (Cu**L**), using H_2_O_2_ as the oxidant, was carried out on thioanisole (**1**) giving sulfoxide enantiomers (**2**) (Scheme 1). The reaction conditions used, including molar ratio between G4 DNA catalyst and Cu**L** cofactor, potassium concentration, buffer pH, and temperature, were those optimized by Mingpan Cheng et al. [11]. In these conditions ([G4 DNA]/[Cu**L**] = 1:5, [KCl] = 150 mM, pH 7.0, 15 °C), the unmodified telomeric G4 DNA catalyzed the reaction with a full conversion and 56% enantioselective excess (ee) [11]. 

All the modified HT21 catalysts, except HT21-ABr1 (93% of conversion), complexed with Cu**L**, exhibited conversion data very similar to the natural one, thus indicating that, in general, the introduction of an unnatural adenosine in the HT21 loop sequence did not reduce the catalytic activities of the evaluated modified G4 DNA. However, data clearly indicated that the presence of modified residues significantly affected the enantioselectivities of the G4-based catalysts (Table 2). Indeed, most of HT21-modified sequences revealed minor enantioselectivities (range of 14–37% ee) compared to the natural one, except in the cases of HT21-AL1 and HT21-AL2. Interestingly, while HT21-AL2 proved able to give an enantiomeric excess (54%) strictly comparable to the unmodified sequence, HT21-AL1 exhibited a remarkably improved enantioselectivity (about 84% ee). This ee value was significantly much higher than that of natural G4 DNA catalyst and, to the best of our knowledge, corresponded to the highest enantioselectivity for DNA based oxidation reaction obtained to date.

These data suggest that, in agreement with those reported for other reactions [9], the three-dimensional structure of HT21, generally preserved in all modified HT21 analogues as indicated by the close similarity of their CD spectra, ensured the notable catalytic performances, giving nearly quantitative conversion. On the other hand, the enantioselectivity of the product largely depended on the loop sequence of the G4 DNA catalyst. Minor modifications in the G4 DNA loop sequence, as the substitution of an A residue with an A^Br^ or A^oxo^, significantly reduced the chiral expression of sulfoxidation reaction. However, the replacement of a natural D-residue with a β-L-2′-deoxyadenosine in a specific loop was proven to be useful to increase the ee value of the reaction, thus improving chiral selectivity. Our data confirmed the key role of the loop regions in close proximity to the terminal G-tetrads for chiral induction of the reactions and expanded the G4 DNA catalytic properties in asymmetric oxidations.

## 3. Materials and Methods

### 3.1. Oligonucleotides’ Synthesis and Purification

The oligonucleotides reported in Table 1 were synthesized on a Millipore Cyclone Plus DNA synthesizer using solid phase β-cyanoethyl phosphoramidite chemistry at 10 µmol scale. The modified monomers were introduced in the sequences using commercially available 5′-dimethoxytrityl-N6-dimethylaminomethylidene-8-bromo-2′-deoxyAdenosine-3′-[(2-cyanoethyl)-(N,N-diisopropyl)]-phosphoramidite and 8-hydroxy-5′-dimethoxytrityl-N6-benzoyldeoxyAdenosine-3′-[(2-cyanoethyl)-(N,N-diisopropyl)]-phosphoramidite (Glen Research, Maravai Life Sciences Inc., San Diego, CA, USA), and β-L-N6-benzoyldeoxyAdenosine-3′-[(2-cyanoethyl)-(N,N-diisopropyl)]-phosphoramidite (ChemGenes Corporation, Wilmington, MA, USA). The oligomers were detached from the support and deprotected by treatment with concentrated aqueous ammonia at room temperature for 24 h (HT21-A^Br^ derivatives) or at 55 °C overnight (all the others). The combined filtrates and washings were concentrated under reduced pressure, redissolved in H_2_O, analyzed, and purified by high-performance liquid chromatography on a Nucleogel SAX column (Macherey-Nagel, 1000-8/46), using buffer A, 20 mM NaH_2_PO_4_/Na_2_HPO_4_ aqueous solution (pH 7.0) containing 20% (*v*/*v*) CH_3_CN, and buffer B, 1 M NaCl, 20 mM NaH_2_PO_4_/Na_2_HPO_4_ aqueous solution (pH 7.0) containing 20% (*v*/*v*) CH_3_CN; a linear gradient from 0 to 100% B for 60 min and a flow rate 1 mL/min were used. The fractions of the oligomers were collected and successively desalted by Sep-pak cartridges (C-18). The isolated oligomers proved to be >98% pure by NMR.

### 3.2. Chemicals

The 4,4′-Bimethyl-2,2′-bipyridine (**L**) and thioanisole were obtained from Fluorochem. Hydrogen peroxide solution (3%), Cu(NO_3_)_2_·3H_2_O, and 3-(N-morpholino)propanesulfonic acid (MOPS) were purchased from Sigma-Aldrich (Merck, St. Louis, MO; USA). The complex Cu**L** was synthesized, as described previously [28], and dissolved in water. **L** (100 mg, 0.54 mmol) was dissolved in ethanol and Cu(NO_3_)_2_·3H_2_O (132 mg, 0.54 mmol) was added. The solution was stirred for 1 h at room temperature until the formation of a blue, solid precipitate. The precipitate was filtered and washed with ethanol and diethyl ether and then dried in an oven (95% yield; ESI-MS: *m*/*z* 309.1 [**L**-Cu^2+^-NO_3_^−^]^+^).

### 3.3. CD Spectroscopy

CD samples of oligonucleotides reported in Table 1 were prepared at an ODN concentration of 20 µM using a 20 mM MOPS buffer (pH 7.0) containing 150 mM KCl and submitted to the annealing procedure (heating at 90 °C and slowly cooling at room temperature). CD spectra of all G-quadruplexes and CD melting curves were registered on a Jasco 715 CD spectrophotometer. For the CD spectra, the wavelength was varied from 220 to 360 nm at a 100-nm min^−1^ scan rate, and the spectra were recorded with a response of 16 s, at a 2.0-nm bandwidth and normalized by subtraction of the background scan with buffer. The temperature was kept constant at 15 °C with a thermoelectrically controlled cell holder (Jasco PTC-348). CD melting curves were registered as a function of the temperature (range: 20 °C–95 °C) for all G-quadruplexes at their maximum Cotton effect wavelengths. The CD data were recorded in a 0.1-cm pathlength cuvette with a scan rate of 0.5 °C/min. Each measurement was the average of three scans.

### 3.4. Oxidation Procedure

DNA (10 µM) was dissolved in MOPS buffer (1 mL, 20 mM, pH 7.0) containing KCl (150 mM), heated for 3 min at 98 °C, and annealed slowly over the course of 2 h to room temperature. An aqueous solution of copper complex Cu**L** (50 µM) was added and stirred for 30 min at 15 °C. Ten µL of solution 0.5 M of thioanisole in CH_3_CN, and subsequently 7.5 µL of 3% H_2_O_2_ aqueous solution were added. The reaction mixture was stirred for 5 h at 15 °C. At the end of the reaction, diethyl ether (3 mL × 3) was added to extract the products. Anhydrous Na_2_SO_4_ was added to the combined organic layers and the solvent was removed under vacuum. The crude products were directly analysed by HPLC using a chiral column. Purification was performed using a Jasco PU-2089 Plus Quaternary Gradient Pump connected to UV-2075 Plus UV/Vis detector (Jasco corporation, Tokyo, Japan) equipped with a Waters Rheodyne injector (Waters Corporation, Milford, MA, USA). HPLC analysis was performed at wavelength 250 nm, using as eluent *n*-hexane and isopropanol (*i*-PrOH) 9:1 and a Lux^®^ Cellulose-1 (Phenomenex, 5 μm, 250 mm × 4.6 mm) column.

## 4. Conclusions

The first DNA-based asymmetric catalysis was described in 2005 [1,2,3,4], thus indicating novel potential uses of DNA as chiral inductor in specific reactions. In this investigation, in order to propose the design of new G4 DNA enantioselective catalysts for sulfoxidation reaction, by using commercially available adenosine derivatives, a series of HT21 analogues were prepared and their catalytic properties were evaluated. In particular, the role of the loop residues in influencing the G4 DNA efficiency in conversion and chiral selection was explored.

The synthesis of HT21 derivatives in Table 1 was very simple and based on standard protocols. CD data strongly suggested that all modified HT21 derivatives adopted G4 structures strictly similar to that of the natural telomeric sequence, despite negligible structural differences probably involving loop regions. CD data indicated that the minor modifications proposed had a very low impact on the main hybrid-type conformation selected in the experimental conditions used. The preserved G4 structure of the human telomeric sequence HT21, induced and stabilized by high potassium concentration, is thought to be closely involved in catalytic activity, since almost all G4 DNA catalysts tested are highly efficient, ensuring complete thioanisole conversion. On the other hand, enantioselectivity data clearly revealed the priority role of loops in inducing product chirality, since minor chemical modifications were enough to influence significantly the enantiomeric excesses obtained. In fact, although the site-specific replacement of loop adenosines with 8-bromo-2′-deoxyadenosine or 8-oxo-2′-deoxyadenosine did not affect appreciably the catalytic activity, it drastically has reduced the chiral selectivity, thus probably suggesting that the modification at C-8 position of adenosine negatively interfered with the microenvironment surrounding the substrate, which is essential to ensure the product chiral induction. On the contrary, the use of L-DNA was confirmed as a promising strategy in modulating the enantioselectivity of a reaction, since the introduction of a single residue with opposite chirality to the rest of the sequence in specific loops consented to obtain ee values strictly comparable or also higher than those shown by the natural sequence. Particularly, the introduction of an A^L^ residue in the first loop was able to induce the highest enantioselectivity (84% ee) for DNA-based sulfoxidation reaction observed to date. Generally, our results support and strongly suggest the use of straightforward chemical modifications for the design and synthesis of novel enantioselective DNA catalysts, thus increasing versatility of asymmetric catalysis. The introduction of L-residues in other positions of the HT21 loop sequence and the application of this approach for the preparation of other G4 DNA catalysts are being evaluated in our laboratory.

## Figures and Tables

**Table 1:** Sequences investigated and their melting temperatures.

Name	Sequence (5′-3′)	Tm °C (±1)
HT21	GGGTTAGGGTTAGGGTTAGGG	69
HT21-ABr1	GGGTTA^Br^GGGTTAGGGTTAGGG	70
HT21-ABr2	GGGTTAGGGTTA^Br^GGGTTAGGG	72
HT21-ABr3	GGGTTAGGGTTAGGGTTA^Br^GGG	71
HT21-AL1	GGGTTA^L^GGGTTAGGGTTAGGG	73
HT21-AL2	GGGTTAGGGTTA^L^GGGTTAGGG	74
HT21-AL3	GGGTTAGGGTTAGGGTTA^L^GGG	68
HT21-Aoxo1	GGGTTA^oxo^GGGTTAGGGTTAGGG	68
HT21-Aoxo2	GGGTTAGGGTTA^oxo^GGGTTAGGG	68
HT21-Aoxo3	GGGTTAGGGTTAGGGTTA^oxo^GGG	68

**Table 2:** Enantioselective oxidation of thioanisole (**1**). * As reported by Mingpan Cheng et al. [11]. All data are repeated for two separate experiments. ^a^ Determined by chiral-phase HPLC within reproducibility of 2%. ^b^ Determined by chiral-phase HPLC within reproducibility of 3%. HPLC chromatograms are reported in Electronic Supplementary Materials.

G4-DNA	%Conversion ^ab^	ee ^b^ %
HT21	99 *	56 *
HT21-AL1	98	84
HT21-AL2	99	54
HT21-AL3	98	36
HT21-Aoxo1	99	37
HT21-Aoxo2	99	14
HT21-Aoxo3	99	21
HT21-ABr1	93	34
HT21-ABr2	98	23
HT21-ABr3	99	28

## Data Availability

The data presented in this study are available on request from the corresponding author.

## Supplementary Materials

The following supporting information can be downloaded at: https://www.mdpi.com/article/10.3390/ijms23031092/s1.
