# Supplementary material for: Discovering New G-Quadruplex DNA Catalysts in Enantioselective Sulfoxidation Reaction
Source: Int J Mol Sci. 2022 Jan 20;23(3):1092. doi: 10.3390/ijms23031092 (PMC8835054; doi:10.3390/ijms23031092)

Supplementary Material

Discovering New G-Quadruplex DNA Catalysts in Enantioselective Sulfoxidation Reaction

Carmen Festa, Veronica Esposito, Daniela Benigno, Simona De Marino, Angela Zampella, Antonella Virgilio * and Aldo Galeone

Department of Pharmacy, University of Naples Federico II, Via D. Montesano 49, I-80131 Naples, Italy; carmen.festa@unina.it (C.F.); verespos@unina.it (V.E.); daniela.benigno@unina.it (D.B.); sidemari@unina.it (S.D.M.); angela.zampella@unina.it (A.Z.); galeone@unina.it (A.G.)
* Correspondence: antonella.virgilio@unina.it; Tel.: +39-081678536

Table of contents

CD melting profiles

HPLC chromatograms

ESI-MS spectrum of CuL complex

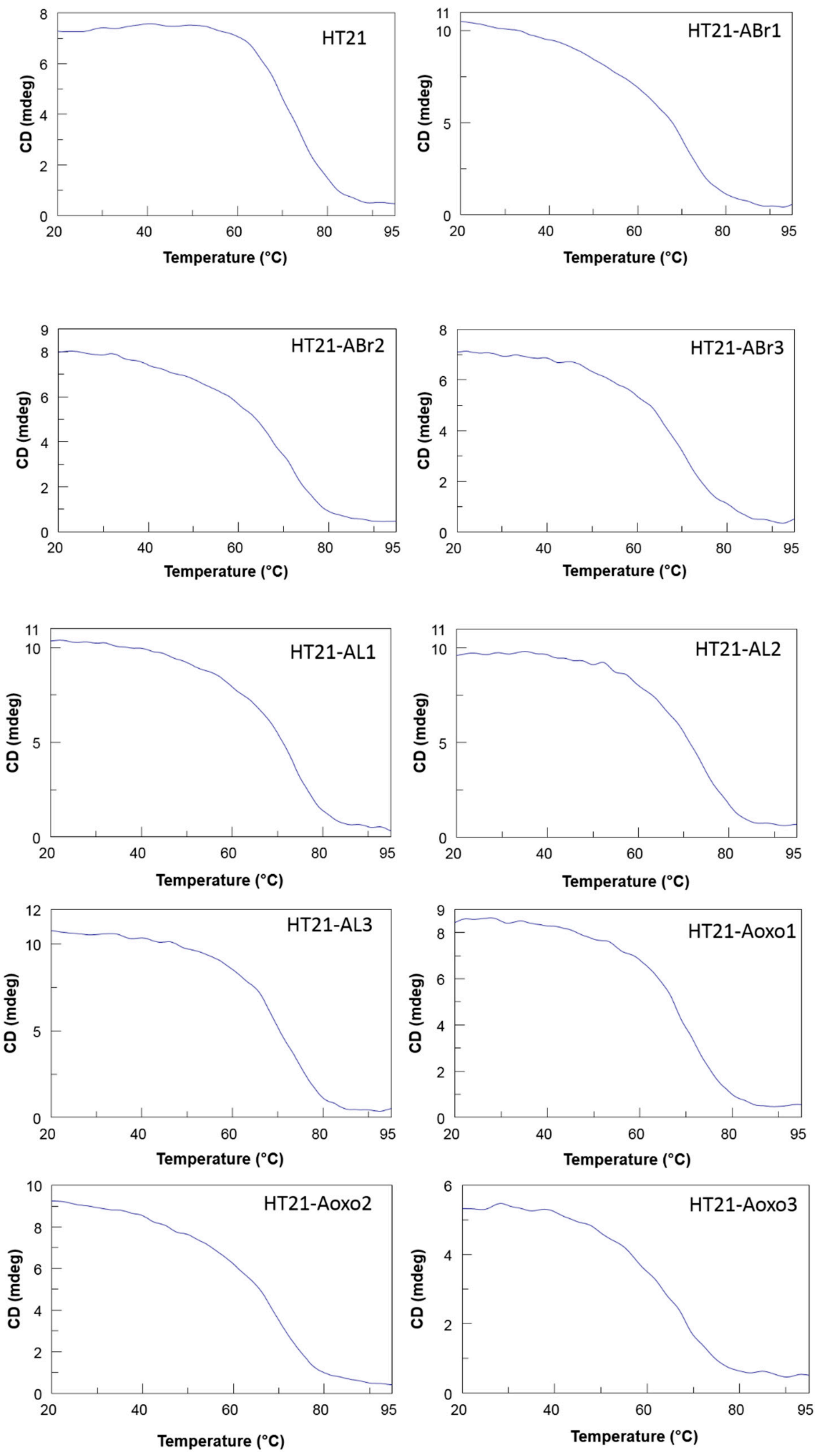

Figure S1. CD melting profiles of analyzed ODNs registered as a function of temperature for all modified quadruplexes at their maximum Cotton effect wavelengths. CD data were recorded in a 0.1 cm pathlength cuvette with a scan rate of 30 °C/h at 20 μM ODN strand concentration in 20 mM MOPS buffer (pH 7.0) containing 150 mM KCl.

HPLC chromatogram 1. Sulfoxidation catalyzed by HT21-AL1•Cu**L**.

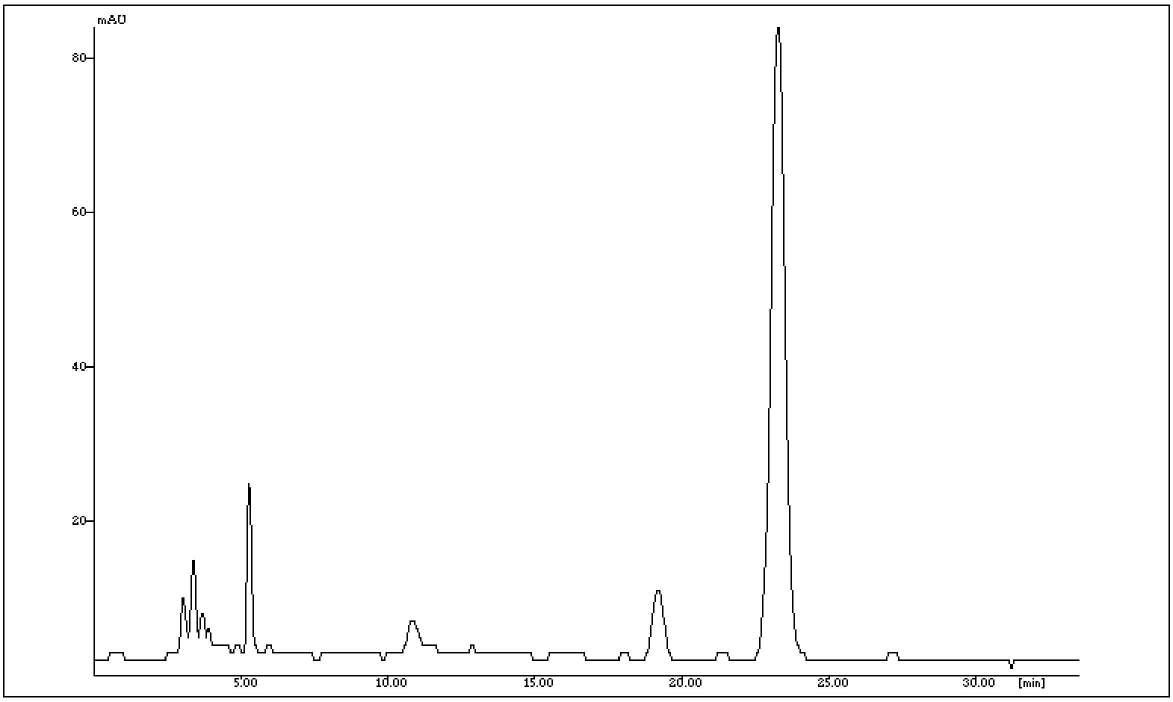

	Peak Name	Rt (Min)	Abs.Wind	%. Wind	Area	Factor	Peak.Group
1	1	3.350	0.10	0.00	71.50	1.00000	
2	2	19.100	0.10	0.00	231.00	1.00000	
3	Unknown	23.242	0.10	0.00	2575.00	1.00000	

HPLC chromatogram 2. Sulfoxidation catalyzed by HT21-AL2•Cu**L**.

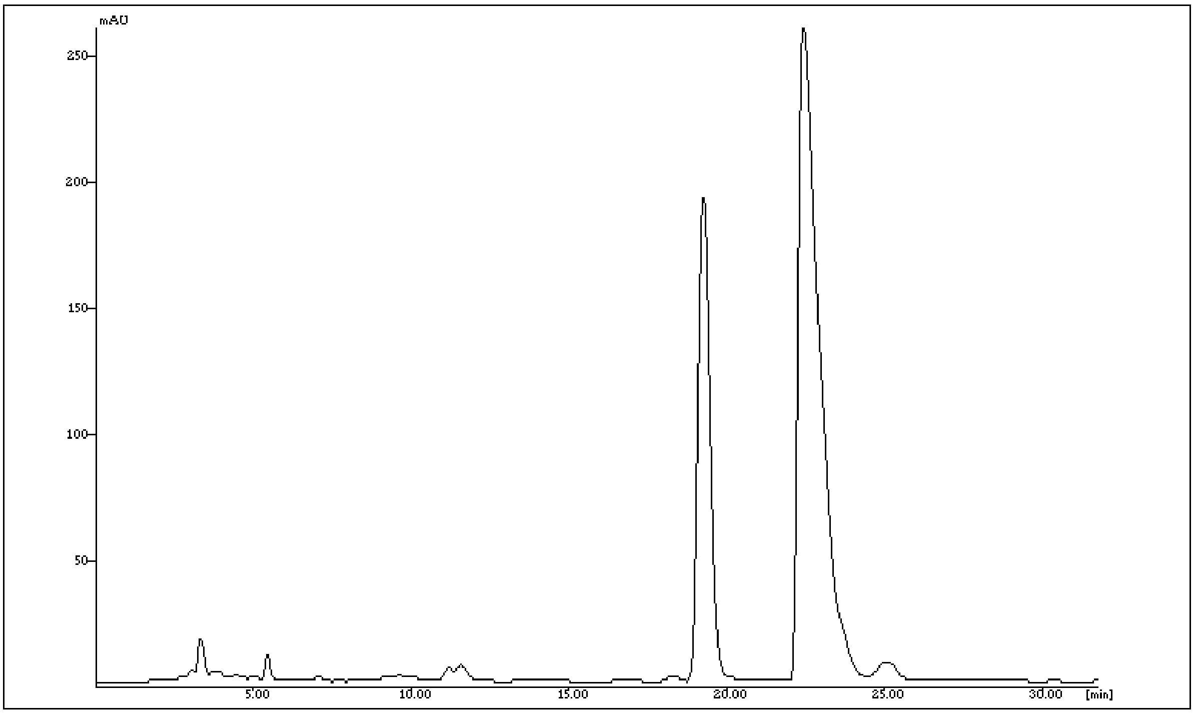

	Peak Name	Rt (Min)	Abs.Wind	%. Wind	Area	Factor	Peak.Group
1	1	3.267	0.10	0.00	137.39	1.00000	
2	2	19.233	0.10	0.00	3547.25	1.00000	
3	2	22.400	0.10	0.00	11721.00	1.00000	

HPLC chromatogram 3. Sulfoxidation catalyzed by HT21-AL3•Cu**L**.

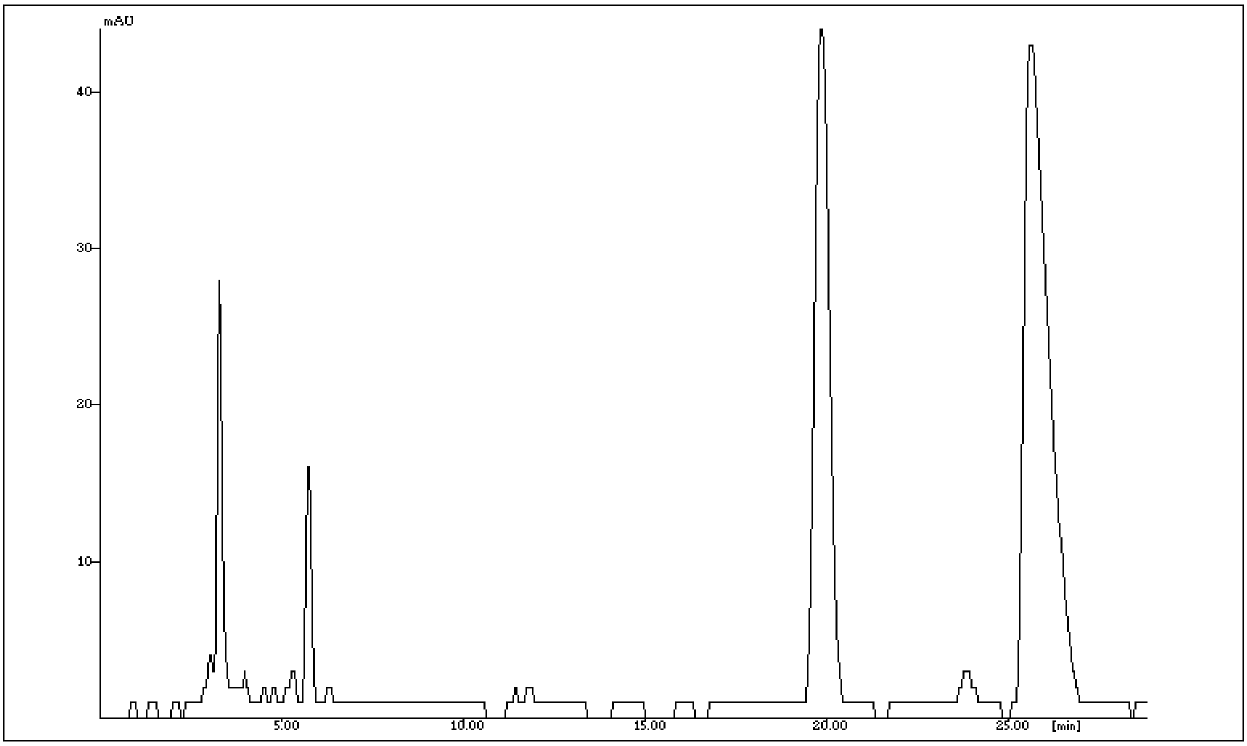

	Peak Name	Rt (Min)	Abs.Wind	%. Wind	Area	Factor	Peak.Group
1	1	3.258	0.10	0.00	177.50	1.00000	
2	2	19.800	0.10	0.00	915.50	1.00000	
3	2	25.558	0.10	0.00	1922.50	1.00000	

HPLC chromatogram 4. Sulfoxidation catalyzed by HT21-Aoxo1•Cu**L**.

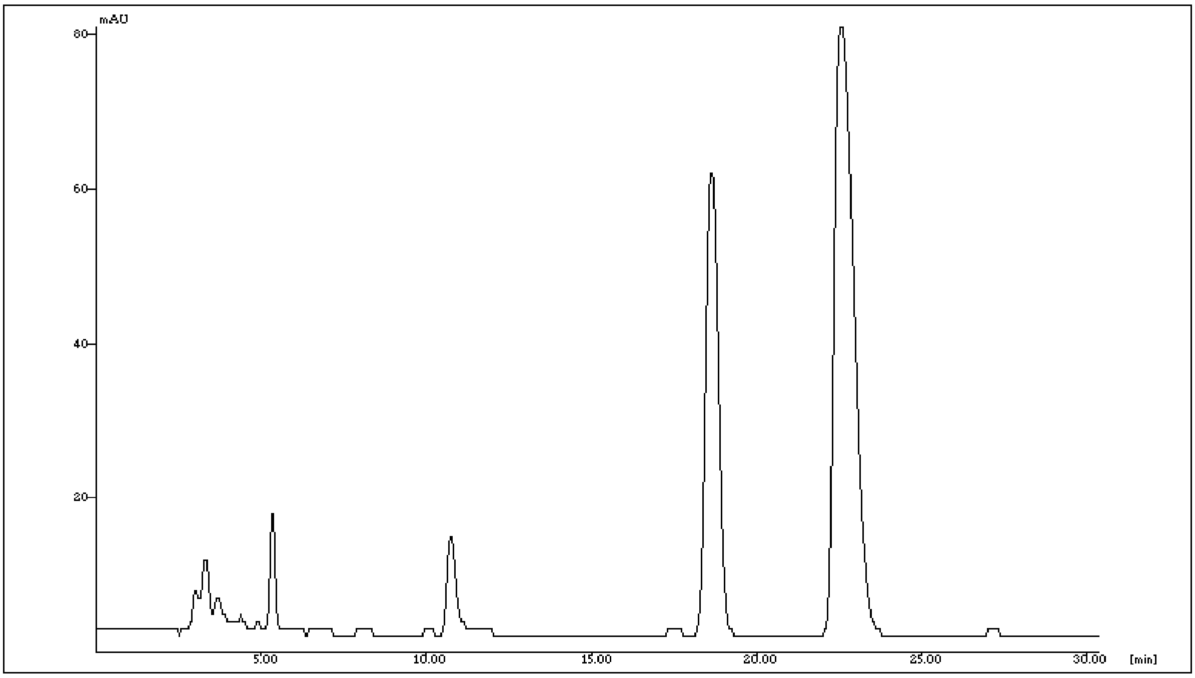

	Peak Name	Rt (Min)	Abs.Wind	%. Wind	Area	Factor	Peak.Group
1	1	3.275	0.10	0.00	58.35	1.00000	
2	2	18.600	0.10	0.00	1445.50	1.00000	
3	2	22.508	0.10	0.00	3052.50	1.00000	

HPLC chromatogram 5. Sulfoxidation catalyzed by HT21-Aoxo2•Cu**L**.

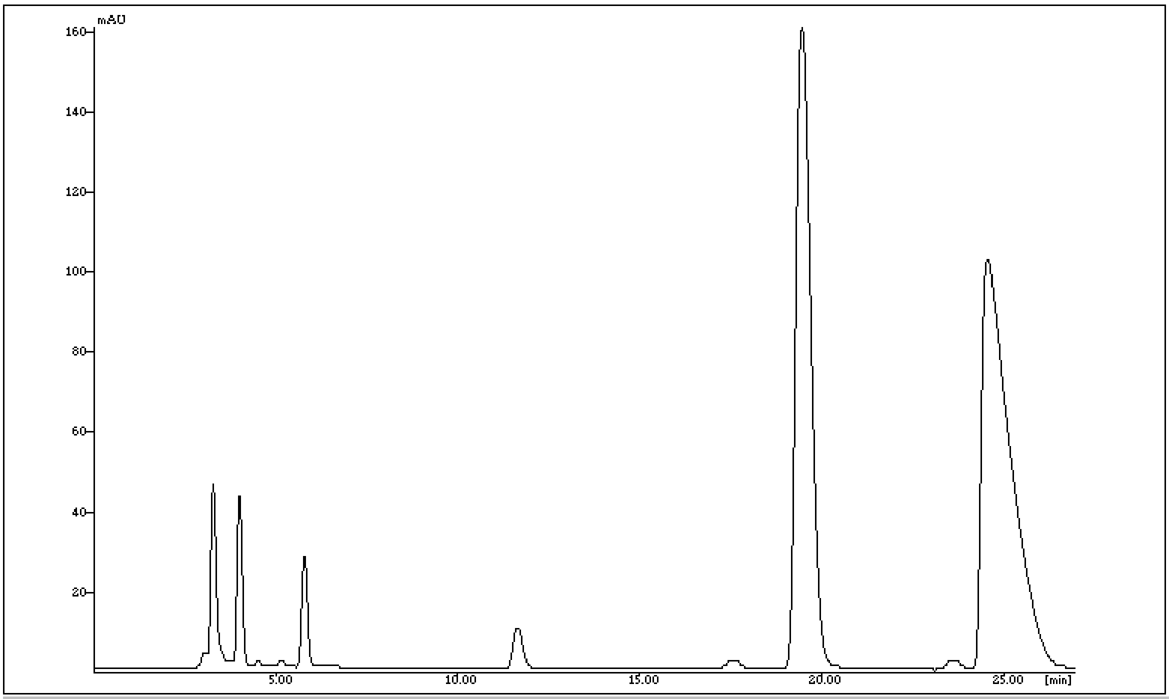

	Peak Name	Rt (Min)	Abs.Wind	%. Wind	Area	Factor	Peak.Group
1	1	3.258	0.10	0.00	330.39	1.00000	
2	2	19.442	0.10	0.00	4153.00	1.00000	
3	2	24.533	0.10	0.00	5321.50	1.00000	

HPLC chromatogram 6. Sulfoxidation catalyzed by HT21-Aoxo3•Cu**L**.

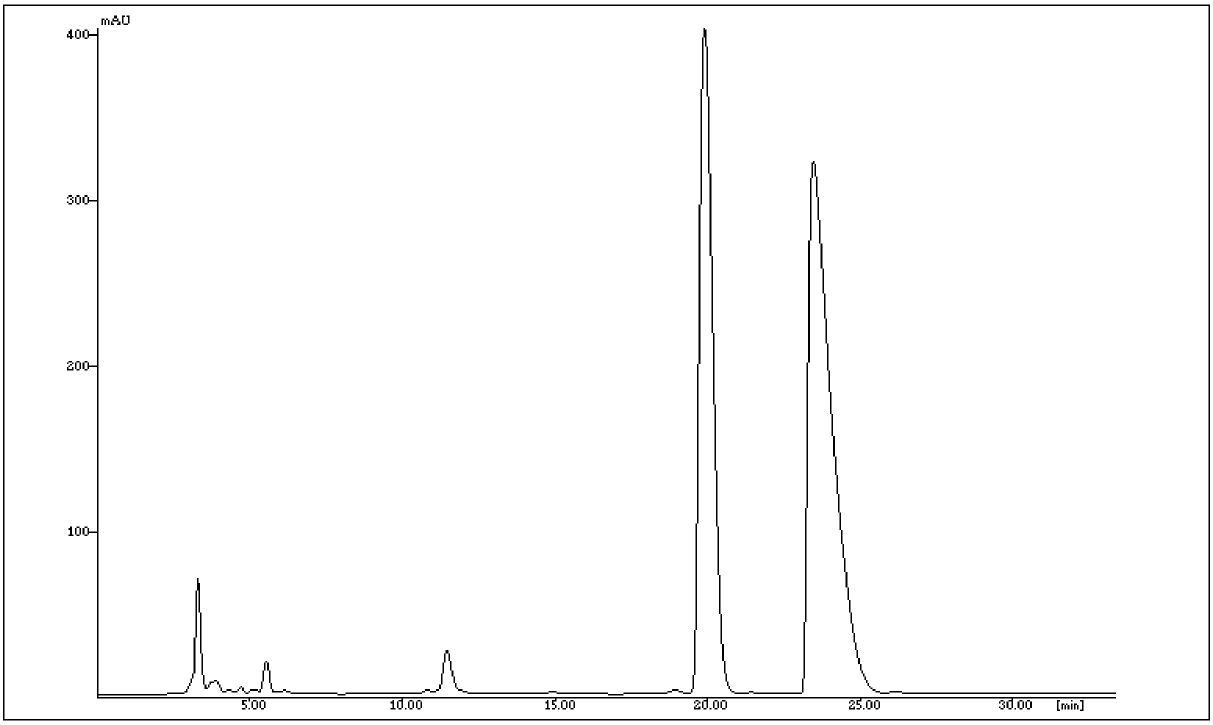

	Peak Name	Rt (Min)	Abs.Wind	%. Wind	Area	Factor	Peak.Group
1	1	3.258	0.10	0.00	595.71	1.00000	
2	2	19.867	0.10	0.00	10887.50	1.00000	
3	2	23.425	0.10	0.00	16300.50	1.00000	

HPLC chromatogram 7. Sulfoxidation catalyzed by HT21-ABr1•Cu**L**.

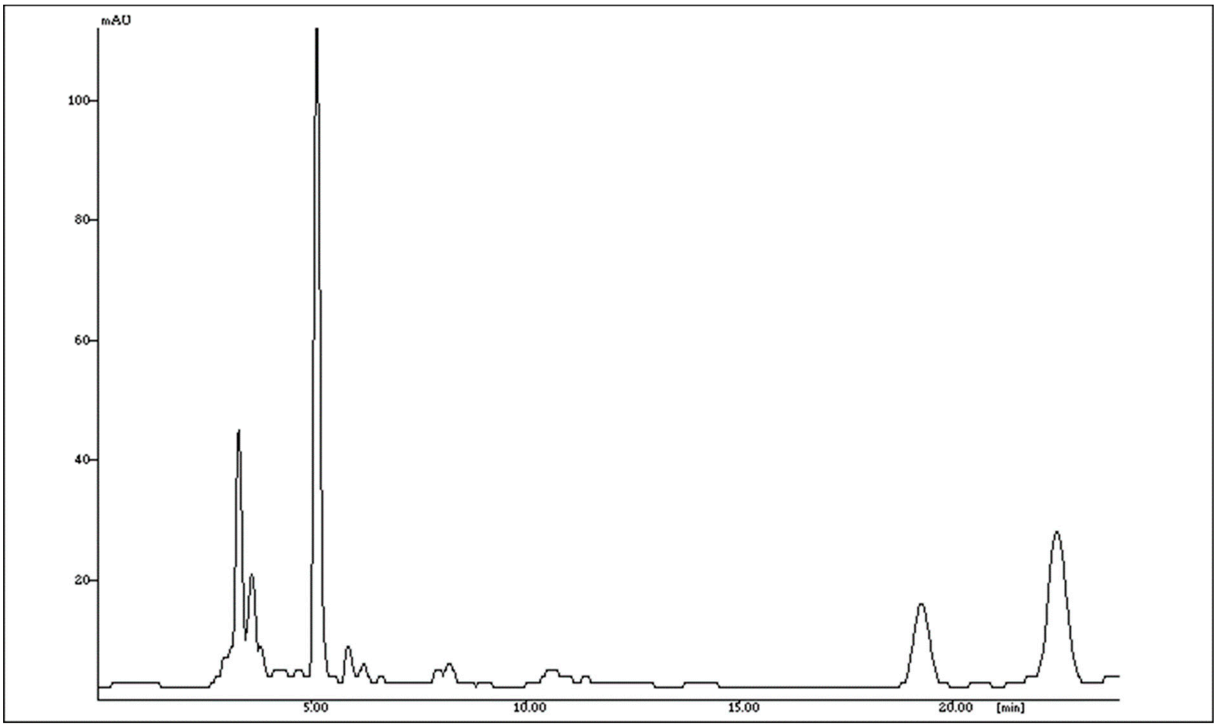

	Peak Name	Rt [Min]	Abs.Wind	%. Wind	Area	Factor	Peak.Group
1	1	3.275	0.10	0.00	263.00	1.00000	
2	2	19.217	0.10	0.00	354.50	1.00000	
3	2	22.375	0.10	0.00	704.50	1.00000	

HPLC chromatogram 8. Sulfoxidation catalyzed by HT21-ABr2•Cu**L**.

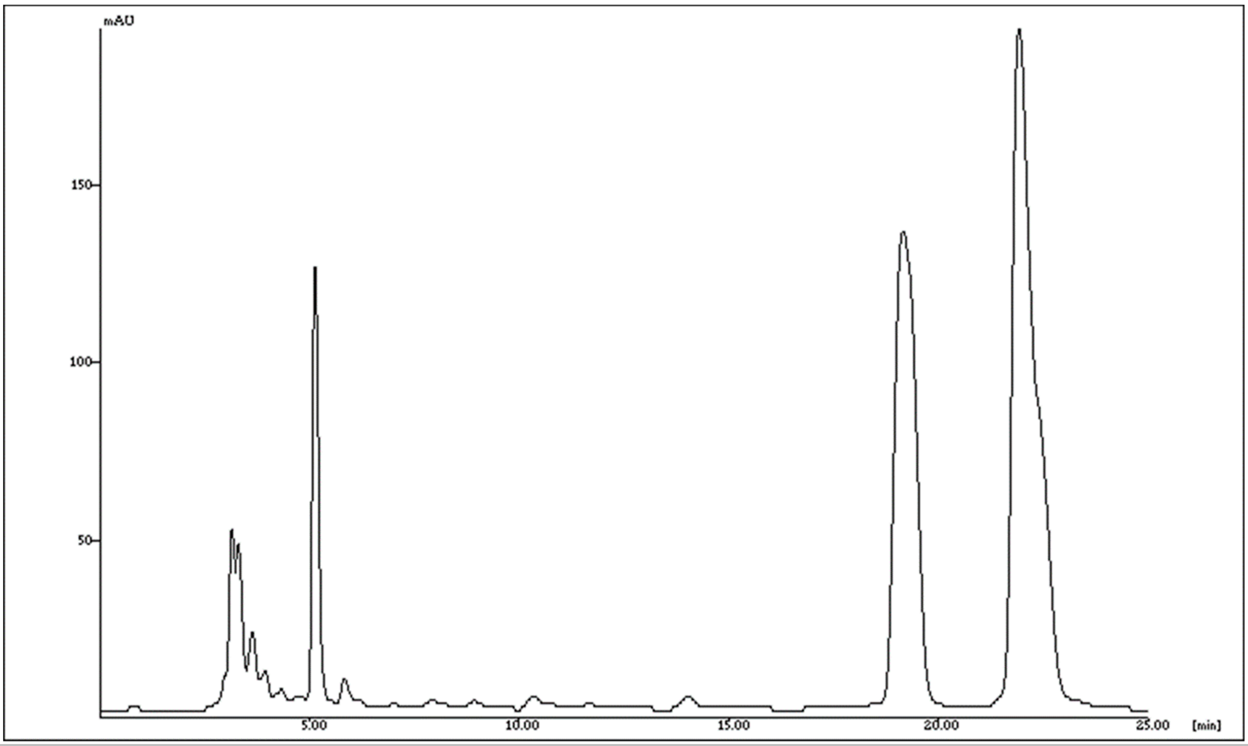

	Peak Name	Rt [Min]	Abs.Wind	%. Wind	Area	Factor	Peak.Group
1	2	3.125	0.10	0.00	686.25	1.00000	
2	2	19.125	0.10	0.00	4409.50	1.00000	
3	2	21.892	0.10	0.00	6915.00	1.00000	

HPLC chromatogram 9. Sulfoxidation catalyzed by HT21-ABr3•Cu**L**.

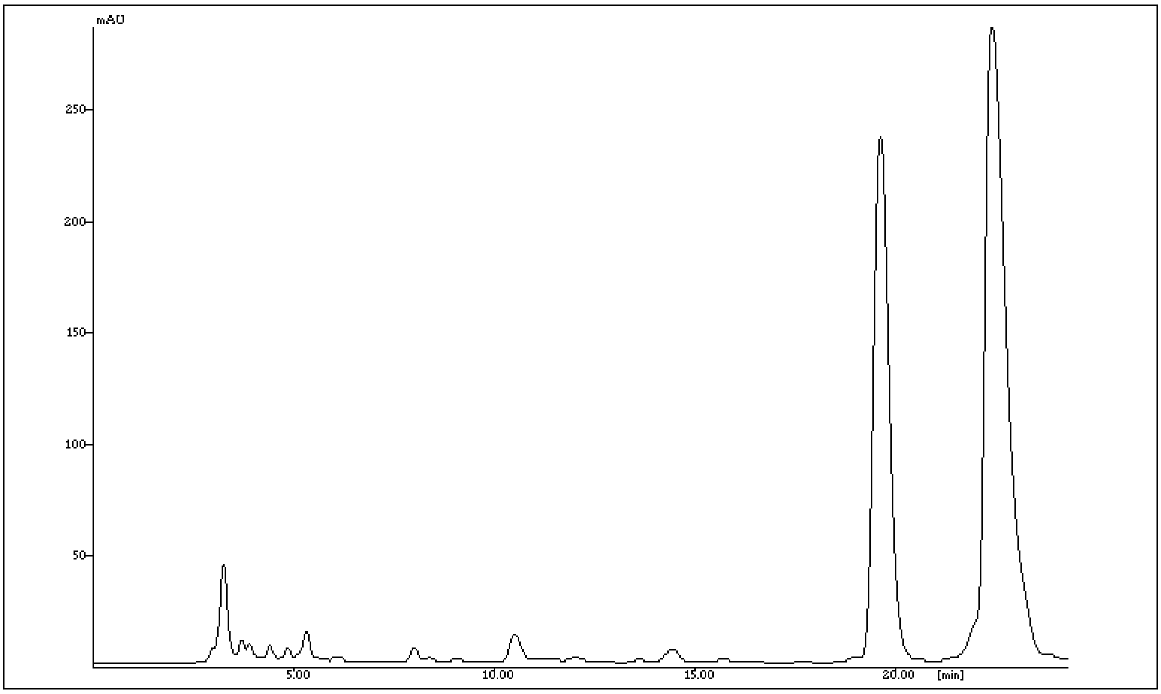

	Peak Name	Rt (Min)	Abs.Wind	%. Wind	Area	Factor	Peak.Group
1	1	3.233	0.10	0.00	473.25	1.00000	
2	2	19.608	0.10	0.00	5675.33	1.00000	
3	2bis	22.392	0.10	0.00	9849.00	1.00000	

ESI-MS spectrum of Cu**L** complex

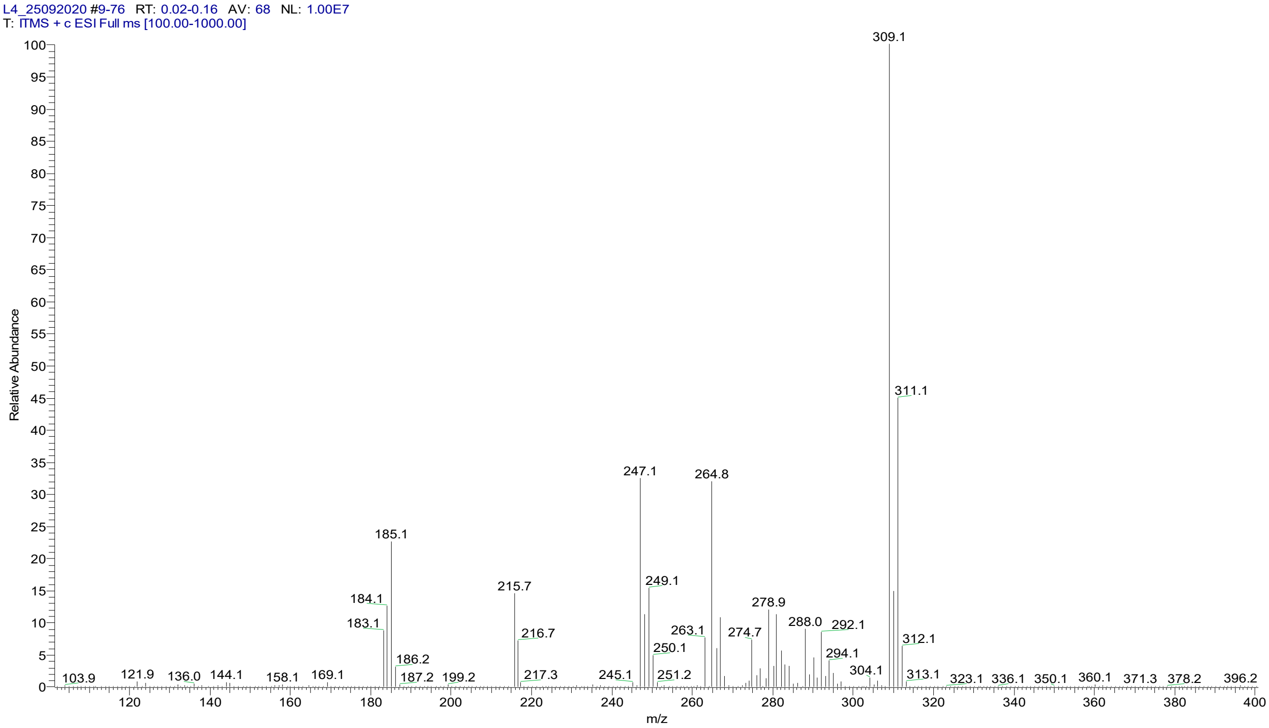

Supplement: Supplementary file 1 [file ijms-23-01092-s001.zip › materials-1519968-supplementary.pdf]
